# Austrian Triad Complicated by Septic Arthritis and Aortic Root Abscess

**DOI:** 10.7759/cureus.3018

**Published:** 2018-07-20

**Authors:** Sandiya Bindroo, Shafinaz Akhter, Kshitij Thakur, Charles Geller

**Affiliations:** 1 Internal Medicine, Crozer Chester Medical Center, Upland, USA; 2 Infectious Disease, Penn Medicine Chester County Hospital, Mount Laurel, USA; 3 Internal Medicine, University of Kentucky College of Medicine, Kentucky, USA; 4 Cardiothoracic Surgery, Crozer Chester Medical Center, Upland, USA

**Keywords:** invasive pneumococcal disease, austrian syndrome, aortic root abscess, septic arthritis, aortic valve endocarditis

## Abstract

Austrian syndrome is a very rare manifestation of invasive Streptococcus pneumoniae infection comprising a triad of pneumonia, meningitis, and endocarditis, also known as Osler’s triad. We herein report a rare case of Austrian syndrome further complicated by septic arthritis.

## Introduction

Austrian syndrome is a rare manifestation of invasive Streptococcus pneumoniae infection. The earliest reports in the literature are from the pre-penicillin era. Currently, in an era of penicillin therapy and pneumococcal conjugate vaccination, diagnosing a disseminated pneumococcal infection is infrequent and rare [1-2].

Austrian syndrome comprises a triad of Streptococcus pneumoniae endocarditis, meningitis, and pneumonia, described by Osler in 1881 [1]. In 1957, Robert Austrian reported a total of eight cases, of which six died secondary to the rupture of the aortic valve [1].

We present a challenging case of Austrian syndrome further complicated by aortic root abscess and septic arthritis in a healthy adult.

## Case presentation

A 60-year-old Caucasian male known to have a bicuspid aortic valve was admitted with a three-day history of cough, altered mental status, and left upper extremity weakness. He did not have any other significant medical or surgical history. At presentation, he was confused and afebrile. His Glasgow Coma Scale (GCS) score was 8/15 (E2V2M4), blood pressure was 124/70 mm Hg, respiratory rate was 22 breaths/min, and oxygen saturation was 86% at room air. He was intubated for airway protection and respiratory support. Cardiac auscultation revealed 3/6 systolic murmur in the right second intercostal space, whereas lung auscultation revealed left lower zone crepitation. The abdominal examination was normal; he did not have any scars to suggest splenectomy. A complete neurologic assessment was not feasible, as the patient was intubated.

The initial laboratory investigations showed a white blood cell count (WBC) of 14.7 K/UL (reference range, 4.0 to 11.0 k/UL) with 90.9% neutrophils, and the platelet count was 34 k/UL (reference range 145-400 k/UL). His erythrocyte sedimentation rate (ESR) was 71 (reference range, 0-22 mm/hr for men). Blood cultures collected before the initiation of antibiotics grew Streptococcus pneumoniae, which was sensitive to ceftriaxone and penicillin. His urine was positive for the Streptococcus pneumoniae antigen.

**Figure 1:**
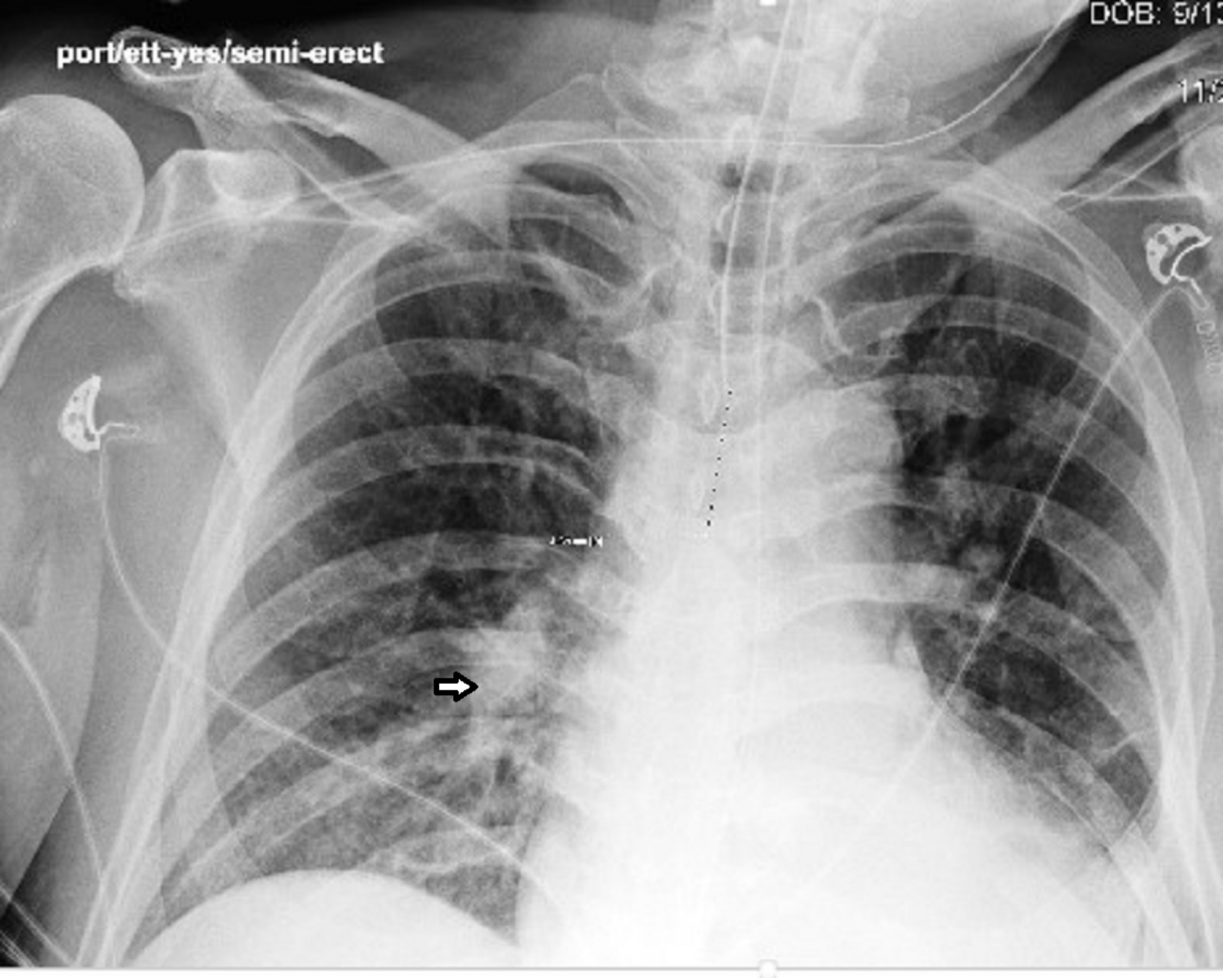
Chest x-ray showing the presence of a left lower lobe infiltrate

A chest radiograph and computerized tomography (CT) scan of the head done on admission demonstrated areas of consolidation over his left lower zone and the dilation of the lateral and third ventricles, respectively (Figure 1).

**Figure 2:**
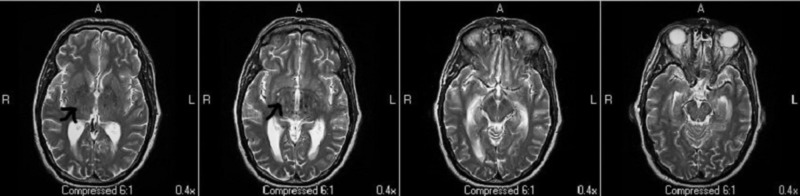
Magnetic resonance imaging (MRI) of the brain showing scattered punctate infarcts in the bilateral cerebrum and very mild hydrocephalus of the third and lateral ventricle

Treatment for bacterial meningitis was initiated with intravenous (IV) ceftriaxone, ampicillin, and dexamethasone empirically. Lumbar puncture was deferred due to the high risk of brain stem herniation secondary to hydrocephalus noted on head CT and high bleeding risk due to thrombocytopenia (platelet count 34 k/UL). On day two of admission, magnetic resonance imaging (MRI) of the brain revealed scattered bilateral cerebral punctate infarcts suggestive of septic emboli along with hydrocephalus (Figure 2). A transesophageal echocardiogram (TEE) revealed caseous calcification of the bicuspid aortic valve and fusion of valve commissures with no vegetation.

Over the course of hospitalization, his condition improved. Antibiotics were de-escalated. On day five of admission, he was successfully extubated. He remained cognitively impaired, which improved gradually.

On day nine of admission, he started complaining of right shoulder pain and had an elevation of leukocyte count (17.2 K/UL) with low-grade fever (99.9F). He underwent right shoulder arthroscopy and debridement for right shoulder septic arthritis. Gram staining of the synovial fluid showed few polymorphs, and the culture was negative. He was subsequently discharged home on a six-week course of IV ceftriaxone 2 g every 24 hours and a plan to get an aortic valve replacement.

Eight weeks after his initial presentation, he had a persistently elevated leukocyte count despite adequate medical management, prompting a treating physician to repeat the echocardiogram. A TEE was performed and documented aortic valve vegetations. Urgent cardiac surgery was performed.

Intraoperative findings include severe pericarditis, a bicuspid aortic valve, and multiple vegetations on the aortic valve leaflet, a 3x3 cm aortic root abscess, and an approximately 10x10 cm retro aortic abscess. He underwent a debridement of the retro-aortic abscess, pericardial patch repair, and aortic valve replacement. A valvular gram stain showed a few polymorphs and negative cultures.

He remained stable in the postoperative period and was subsequently discharged on intravenous penicillin to complete a course for another six weeks.

On a follow-up two months after discharge home, he remained stable with no significant anatomic or functional heart abnormalities and no neurological deficits.

## Discussion

Streptococcus pneumoniae (S. pneumoniae) is a typical example of a highly invasive, Gram-positive, extracellular bacterial pathogen [3]. In the antibiotic era, Streptococcus pneumoniae endocarditis is responsible for less than 3% of all cases of endocarditis in native valves. Pneumococcal endocarditis often causes extensive valve destruction and paravalvular involvement with a high mortality rate of 50% to 62% without surgical treatment [4].

Pneumococcal endocarditis is seen more commonly in middle-aged, debilitated men with a history of chronic alcoholism. Other risk factors for invasive pneumococcal infections include extremes of age; diabetes mellitus; chronic renal insufficiency; chronic liver disease; chronic pulmonary disease; anatomical or functional asplenia; tobacco abuse; certain ethnic groups, such as Alaskan natives; and other immunosuppressive conditions, such as human immunodeficiency virus (HIV) and multiple myeloma [5]. Interestingly, our case fitted in none of these predisposing conditions.

Classically, an invasive pneumococcal infection is meningitis and bacteremia and is defined as an infection confirmed by the isolation of Streptococcus pneumoniae from a normally sterile site (e.g., blood or cerebrospinal fluid but not sputum) [3]. In our case, the clinical diagnosis was confirmed by the positive blood culture and the presence of the Streptococcus pneumoniae antigen in urine. Though we were initially unable to perform a lumbar puncture secondary to increased bleeding risk, it was later on deemed unnecessary, as the patient improved clinically with medical management.

A clinical challenge exists in the early recognition of cardiac involvement, especially in patients who typically lack the peripheral features of infective endocarditis. A transesophageal echocardiogram (TEE) is significantly more sensitive than a transthoracic echocardiogram (TTE) in the diagnosis of endocarditis [6]. In our patient, the initial TEE was negative, delaying the confirmation of diagnosis further but high clinical suspicion guided the rest of the clinical course. This highlights the importance of careful daily review and clinical examination and maintaining a low threshold for suspecting endocarditis in a patient with ongoing sepsis.

Moreover, the involvement of different organs in patients with pneumococcal bacteremia also confounds the clinical presentation. It is important to be mindful of the potential for the spread of S. pneumoniae to other distant sites [7]. Septic arthritis is an uncommon complication of pneumococcal bacteremia. We herein report the presence of septic arthritis.

In a retrospective study of 80 cases of pneumococcal meningitis, only six patients developed endocarditis and death occurred in two patients due to cardiogenic shock [8]. However, Aronin et al. reported a 42% prevalence of Osler’s triad in a review of pneumococcal endocarditis in the penicillin era, with a mortality rate greater than 50% [9].

Current literature on the treatment of Austrian syndrome emphasizes combined medical and surgical intervention. Due to the emergence of penicillin-resistant isolates, the initial use of combination antibiotics, such as a third-generation cephalosporin and vancomycin, are recommended. The antibiotic regimen can be modified once antimicrobial susceptibilities are available. Early surgical intervention in severe cases with invasive pneumococcal endocarditis decreases mortality by approximately 50% [4,10].

## Conclusions

Given the rarity and high mortality associated with this disease, the report serves as a reminder of rare clinical illness and highlights the importance of early diagnosis and appropriate treatment to reduce the associated complications. Through this report, we wish to illustrate the importance of maintaining a high index of suspicion and pursuing this diagnosis in everyone who presents with severe sepsis and suspected pneumococcal infection, even if they do not have predisposing risk factors.
